# Supplementary material for: Molecular basis for gating of cardiac ryanodine receptor explains the mechanisms for gain- and loss-of function mutations
Source: Nat Commun. 2022 May 20;13:2821. doi: 10.1038/s41467-022-30429-x (PMC9123176; doi:10.1038/s41467-022-30429-x)
Supplement: Supplementary file 1 — Supplementary Information [file 41467_2022_30429_MOESM1_ESM.pdf]

Supplementary Information for

Molecular basis for gating of cardiac ryanodine receptor explains the mechanisms for gain- and loss-of function mutations

Takuya Kobayashi[1,5], Akihisa Tsutsumi[2,5], Nagomi Kurebayashi[1], Kei Saito[3], Masami Kodama[1], Takashi Sakurai[1], Masahide Kikkawa[2], Takashi Murayama[1,*] and Haruo Ogawa[4,6,*]

[1]Department of Cellular and Molecular Pharmacology, Juntendo University Graduate School of Medicine, Tokyo, Japan

[2]Department of Cell Biology and Anatomy, Graduate School of Medicine, The University of Tokyo, Tokyo, Japan

[3]Department of Life Sciences, Graduate School of Arts and Sciences, The University of Tokyo, Tokyo, Japan

[4]Department of Structural Biology, Graduate School of Pharmaceutical Sciences, Kyoto University, Japan

[5]These authors contributed equally

[6]Lead Contact

*Correspondence: Email: takashim@juntendo.ac.jp (T.M.); haru@pharm.kyoto-u.ac.jp (H.O.)

Items contained in this file:

Supplementary Figures 1-12
Supplementary Table 1-2
Supplementary References

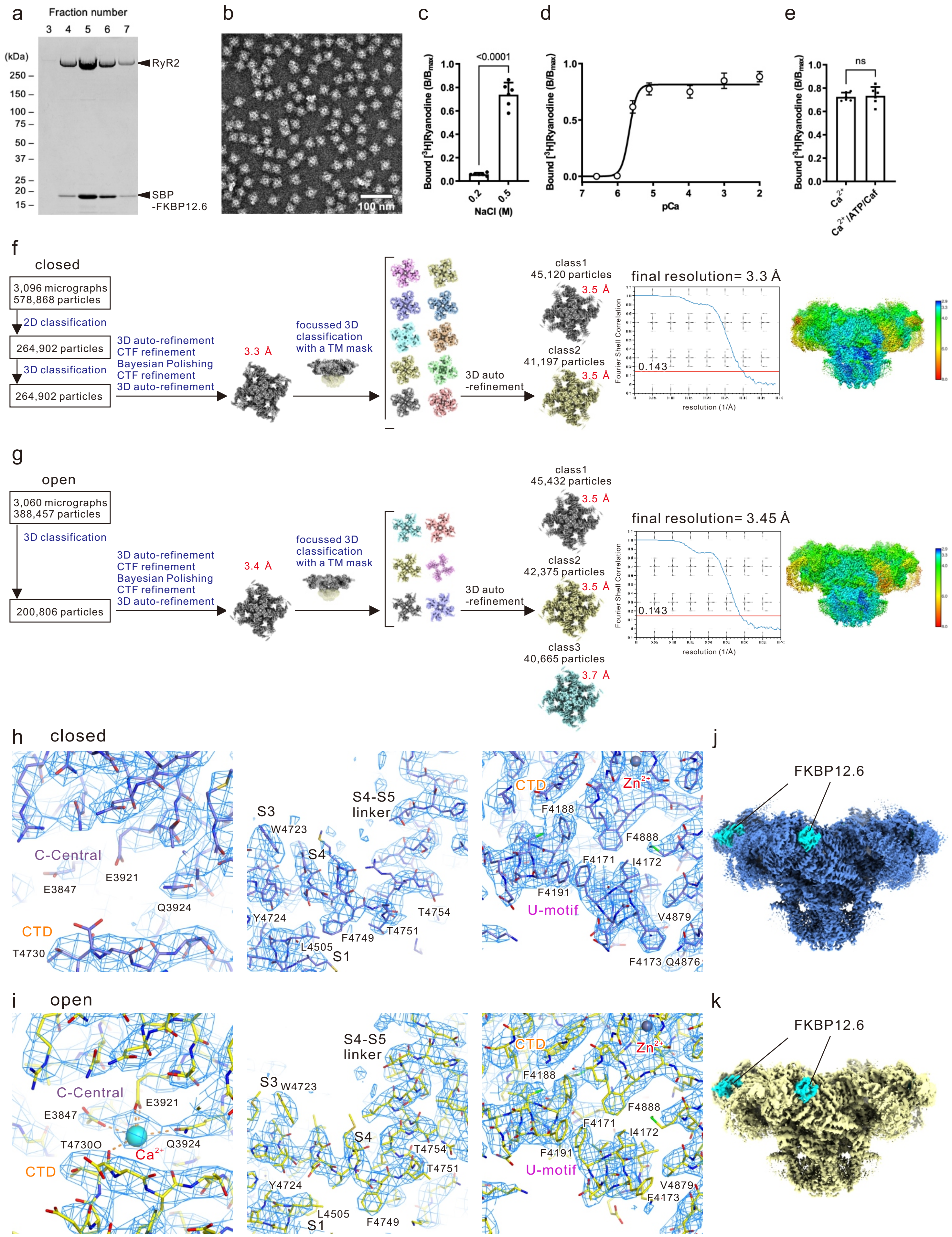
a
Fraction number
3 4 5 6 7
(kDa)
250
150
100
75
50
37
25
20
15
RyR2
SBP
-FKBP12.6
b
100 nm
c
<0.0001
Bound [3H]Ryanodine (B/Bmax)
NaCl (M)
d
pCa
e
ns
Ca2+
Ca2+/ATP/Caf
f
closed
3,096 micrographs
578,868 particles
2D classification
264,902 particles
3D classification
264,902 particles
3D auto-refinement
CTF refinement
Bayesian Polishing
CTF refinement
3D auto-refinement
3.3 Å
focussed 3D
classification
with a TM mask
3D auto
-refinement
class1
45,120 particles
3.5 Å
class2
41,197 particles
3.5 Å
final resolution= 3.3 Å
Fourier Shell Correlation
0.143
resolution (1/Å)
g
open
3,060 micrographs
388,457 particles
3D classification
200,806 particles
3D auto-refinement
CTF refinement
Bayesian Polishing
CTF refinement
3D auto-refinement
3.4 Å
focussed 3D
classification
with a TM mask
3D auto
-refinement
class1
45,432 particles
3.5 Å
class2
42,375 particles
3.5 Å
class3
40,665 particles
3.7 Å
final resolution= 3.45 Å
Fourier Shell Correlation
0.143
resolution (1/Å)
h
closed
C-Central
E3847
E3921
Q3924
CTD
T4730
S3
W4723
S4-S5
linker
S4
Y4724
T4754
F4749
T4751
L4505
S1
CTD
Zn2+
F4188
F4888
F4171
I4172
F4191
U-motif
V4879
F4173
Q4876
j
FKBP12.6
i
open
C-Central
E3921
E3847
T4730O
Q3924
Ca2+
CTD
S4-S5
linker
S3
W4723
S4
T4754
T4751
Y4724
L4505
S1
F4749
CTD
Zn2+
F4188
F4888
F4171
I4172
F4191
U-motif
V4879
F4173
k
FKBP12.6

Supplementary Figure 1. Single particle analysis of recombinant mouse RyR2 on the closed and open states. **a** SDS-PAGE of the peak fractions of StrepTrap column chromatography. Data are the representative of five independent experiments. **b** EM image of the purified RyR2 complex by negative staining. Data are the representative of three independent experiments. **c** [^{3}H]Ryanodine binding of the purified RyR2 in the presence of 100 μM Ca^{2+} at 0.2 M and 0.5 M NaCl. Data are shown as means ± SD (n = 6) and were analyzed by one-way ANOVA with Dunnett's test. **d** Ca^{2+} dependent [^{3}H]ryanodine binding of the purified RyR2 in 0.5 M NaCl solution. Data are shown as means ± SD (n = 6). **e** [^{3}H]Ryanodine binding of the purified RyR2 in 0.5 M NaCl solution with Ca^{2+} (100 μM) and Ca^{2+} plus ATP (5 mM)/caffeine (10 mM). Data are shown as means ± SD (n = 6) and were analyzed by one-way ANOVA with Dunnett's test. **f**, **g** Workflows for cryo-EM data processing and estimations of resolution by Fourier shell correlation (FSC) plots and local resolution EM maps in the presence of EGTA (closed state, **f**) and in the presence of 100 μM Ca^{2+} (open state, **g**), respectively. **h**, **i** Density maps for the reconstructed structures in the presence of EGTA (closed state, **h**) and in the presence of 100 μM Ca^{2+} (open state, **i**), respectively. Left, around the Ca^{2+} binding site, contour level at 0.03. Center, around the S4-S5 linker, contour level at 0.035. Right, around the U-motif, contour levels for the closed and open states were 0.035 and 0.03, respectively. **j**, **k** Overall density maps of the closed (**i**) and open (**j**) states with FKBP12.6. The density corresponding to FKBP12.6 is colored with cyan. Contour levels for the closed and open states were 0.023 and 0.017, respectively. Source data are provided as a Source Data file.

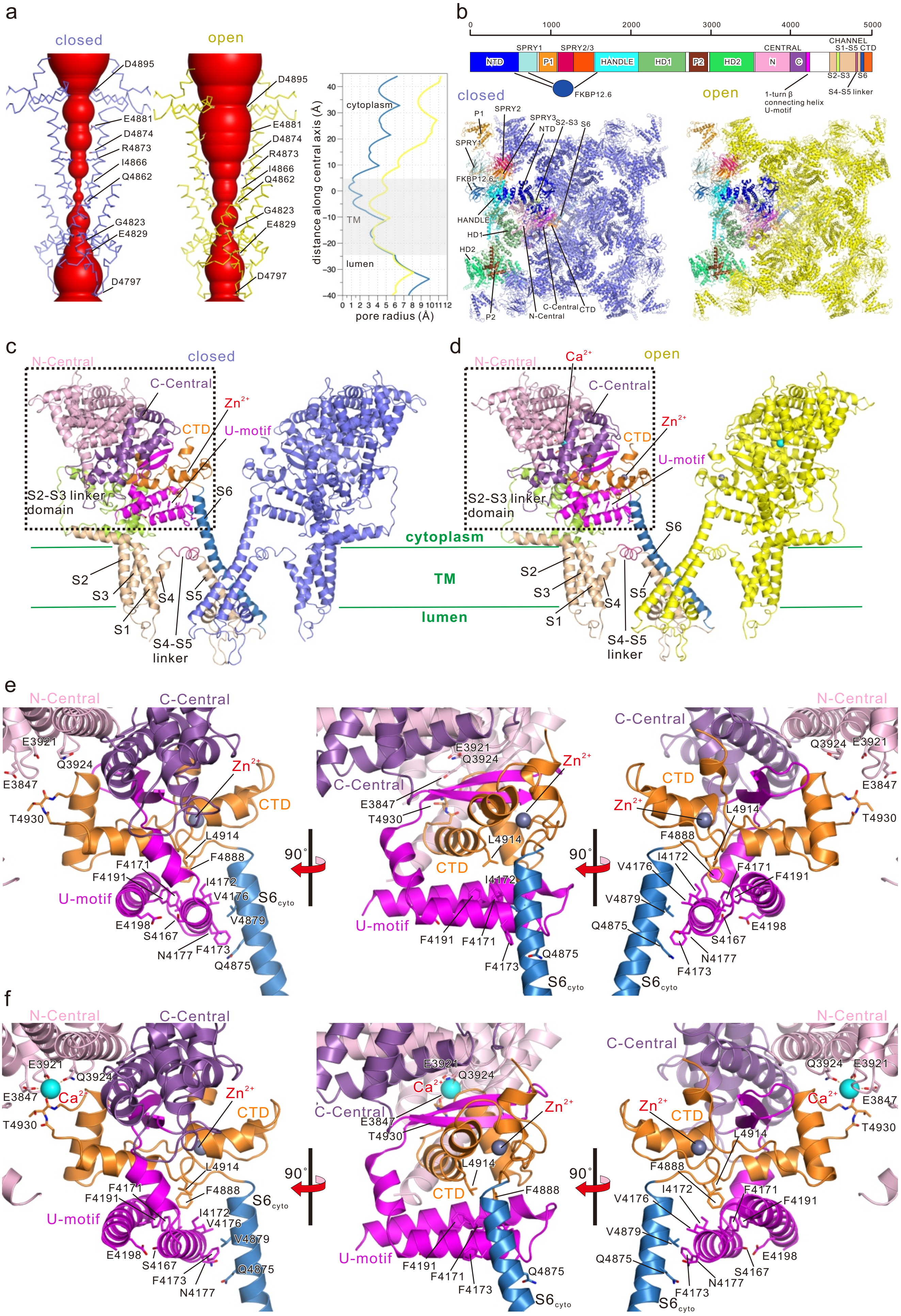
a
closed
open
D4895
E4881
D4874
R4873
I4866
Q4862
G4823
E4829
D4797
cytoplasm
TM
lumen
distance along central axis (Å)
pore radius (Å)
b
0
1000
2000
3000
4000
5000
NTD
SPRY1
P1
SPRY2/3
HANDLE
HD1
P2
HD2
CENTRAL
N
C
CHANNEL
S1-S5 CTD
S2-S3
S6
S4-S5 linker
FKBP12.6
1-turn β
connecting helix
U-motif
closed
open
SPRY1
SPRY2
SPRY3
NTD
S2-S3
S6
FKBP12.6
HANDLE
HD1
HD2
P2
N-Central
C-Central
CTD
c
N-Central
closed
C-Central
Zn2+
CTD
U-motif
S2-S3 linker
domain
S6
S2
S3
S4
S5
S1
S4-S5
linker
cytoplasm
TM
lumen
d
N-Central
Ca2+
open
C-Central
Zn2+
CTD
U-motif
S2-S3 linker
domain
S6
S2
S3
S4
S5
S1
S4-S5
linker
e
N-Central
C-Central
E3921
Q3924
E3847
T4930
Zn2+
CTD
L4914
F4888
F4171
F4191
I4172
V4176
U-motif
S6cyto
V4879
E4198
S4167
N4177
F4173
Q4875
90°
f
N-Central
C-Central
E3921
Q3924
E3847
Ca2+
T4930
Zn2+
CTD
L4914
F4888
F4171
F4191
I4172
V4176
U-motif
S6cyto
V4879
E4198
S4167
F4173
N4177
Q4875
90°

Supplementary Figure 2. Details of the structures of RyR2. a Channel pore and pore radii along the ion conducting pathway of the closed and open states of RyR2 calculated by HOLE [1]. **b** Top, domain organization of rat RyR2 protomer. Bottom, structures of RyR2 viewed from cytoplasmic side (top view) in the closed state (left) and open state (right). One of four protomers in each state is colored, and the color of each domain follows the color shown in top. **c**, **d** Ribbon model of core domains of RyR2 in the closed (**c**) and open (**d**) states. In the left protomer, each domain is colored (N-Central, light pink; C-Central, purple; U-motif, magenta; S1-S5, wheat; S2-S3 linker domain, light green; S4-S5 linker, warm pink; S6, blue; CTD, orange). Ca^{2+}, shown as cyan ball; Zn^{2+}, shown as gray ball. **e** and **f** Magnified views of the dotted box in (**c**) and (**d**), respectively. Only N-/C-Central, U-motif, $S6_{cyto}$, and CTD are shown. The middle and right were rotated 90° and 180° to the left, respectively. Ca^{2+}-binding site is composed of the carbonyl oxygen of T4930 (CTD) from the lower side and the side chains (E3847, E3921, and Q3924) from N-Central.

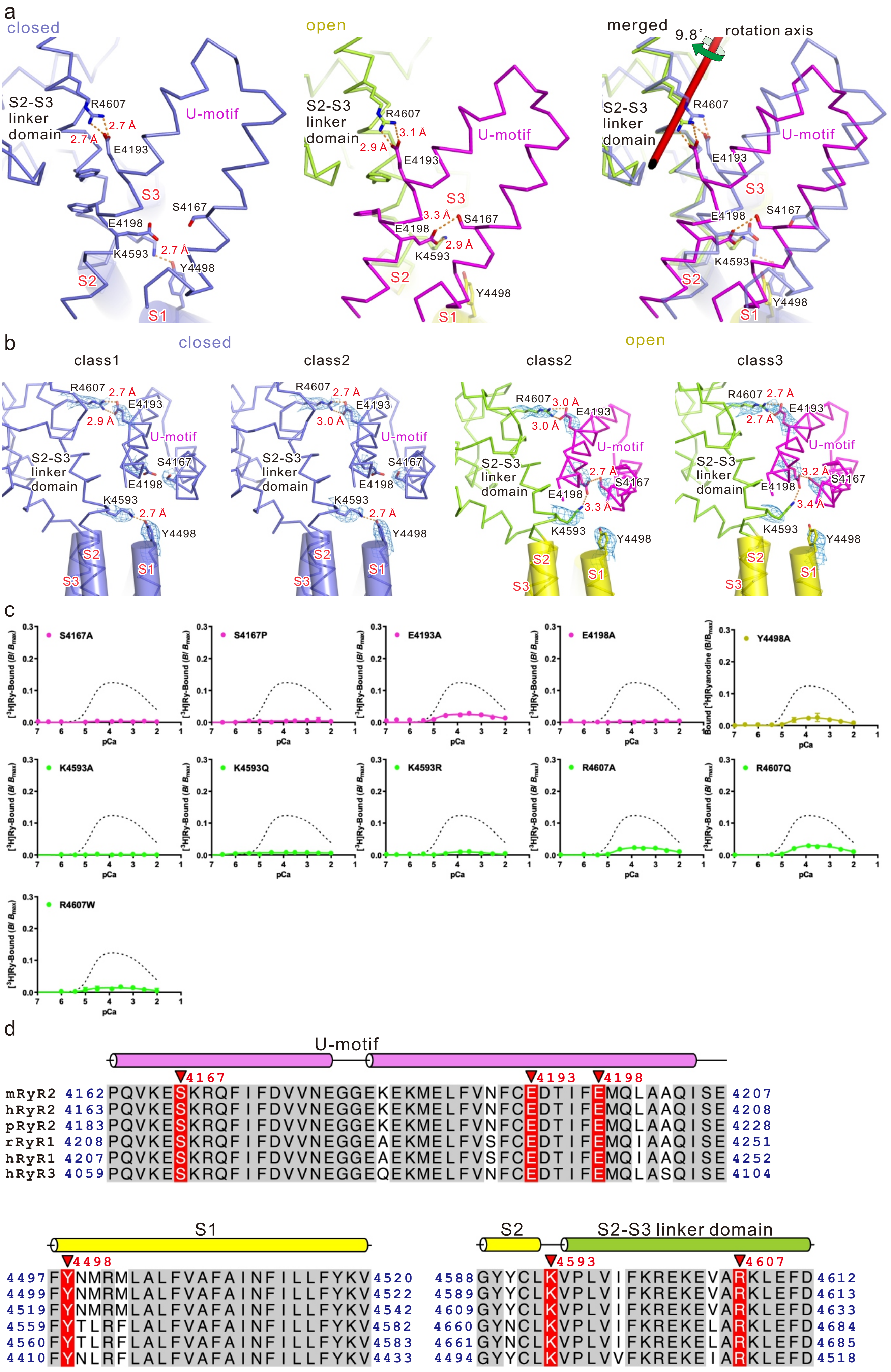
a
closed
open
merged
9.8°
rotation axis
S2-S3 linker domain
R4607
2.7 Å
E4193
U-motif
S3
S4167
E4198
K4593
Y4498
S2
S1
3.1 Å
2.9 Å
3.3 Å
b
class1
class2
class3
3.0 Å
3.2 Å
3.4 Å
c
S4167A
S4167P
E4193A
E4198A
Y4498A
K4593A
K4593Q
K4593R
R4607A
R4607Q
R4607W
[3H]Ry-Bound (B/ Bmax)
Bound [3H]Ryanodine (B/Bmax)
pCa
d
U-motif
▼4167 ▼4193 ▼4198
mRyR2 4162 PQVKESKRQFIFDVVNEGGEKEKMELFVNFCEDTIFEMQLAAQISE 4207
hRyR2 4163 PQVKESKRQFIFDVVNEGGEKEKMELFVNFCEDTIFEMQLAAQISE 4208
pRyR2 4183 PQVKESKRQFIFDVVNEGGEKEKMELFVNFCEDTIFEMQLAAQISE 4228
rRyR1 4208 PQVKESKRQFIFDVVNEGGEAEKMELFVSFCEDTIFEMQIAAQISE 4251
hRyR1 4207 PQVKESKRQFIFDVVNEGGEAEKMELFVSFCEDTIFEMQIAAQISE 4252
hRyR3 4059 PQVKESKRQFIFDVVNEGGEQEKMELFVNFCEDTIFEMQLASQISE 4104
S1
▼4498
4497 FYNMRMLALFVAFAINFILLFYKV 4520
4499 FYNMRMLALFVAFAINFILLFYKV 4522
4519 FYNMRMLALFVAFAINFILLFYKV 4542
4559 FYTLRFLALFVAFAINFILLFYKV 4582
4560 FYTLRFLALFVAFAINFILLFYKV 4583
4410 FYNLRFLALFVAFAINFILLFYKV 4433
S2
S2-S3 linker domain
▼4593 ▼4607
4588 GYYCLKVPLVIFKREKEVARKLEFD 4612
4589 GYYCLKVPLVIFKREKEVARKLEFD 4613
4609 GYYCLKVPLVIFKREKEVARKLEFD 4633
4660 GYNCLKVPLVIFKREKELARKLEFD 4684
4661 GYNCLKVPLVIFKREKELARKLEFD 4685
4494 GYYCLKVPLVVFKREKEIARKLEFD 4518

Supplementary Figure 3. U-motif/S2-S3 linker domain interactions and functional analysis of the related mutants. a Interface of the U-motif and S2-S3 linker domain in the closed state (left), open state (center), and overlay of both states (right) shown as a Cα model viewed from cytoplasmic side. Amino acid residues involved in the key interactions are shown as stick models. The color of carbon atoms is the same as that of Cα; oxygen, red; nitrogen, blue. The TM region is indicated in yellow and the region forming α-helices is overlaid with the cylinder model. Hydrogen bonds are shown as orange dotted lines. **b** Interface of U-motif and S2-S3 linker domain in the closed state or open state in the different classes in the same views shown in Fig. 2a and 2b. Density maps around side chains are superimposed and contoured at 0.025. **c** Functional analysis of mutants involved in the U-motif/S2S3 domain interaction. Ca^{2+}-dependent [^{3}H]ryanodine binding of WT (dotted line) and individual mutants. Data are shown as means ± SD (n = 4). **d** Multiple sequence alignment of three RyR isoforms around the U-motif, S1, and S2 to the initial S2-S3 linker domain. The gray-shaded residues are the identical sequences among isoforms, and the residues shown in red-shade are the residues for which the mutants were prepared and the functional assays were performed. Secondary structures are shown above the alignment. m, mouse; h, human; p, pig; r, rabbit.

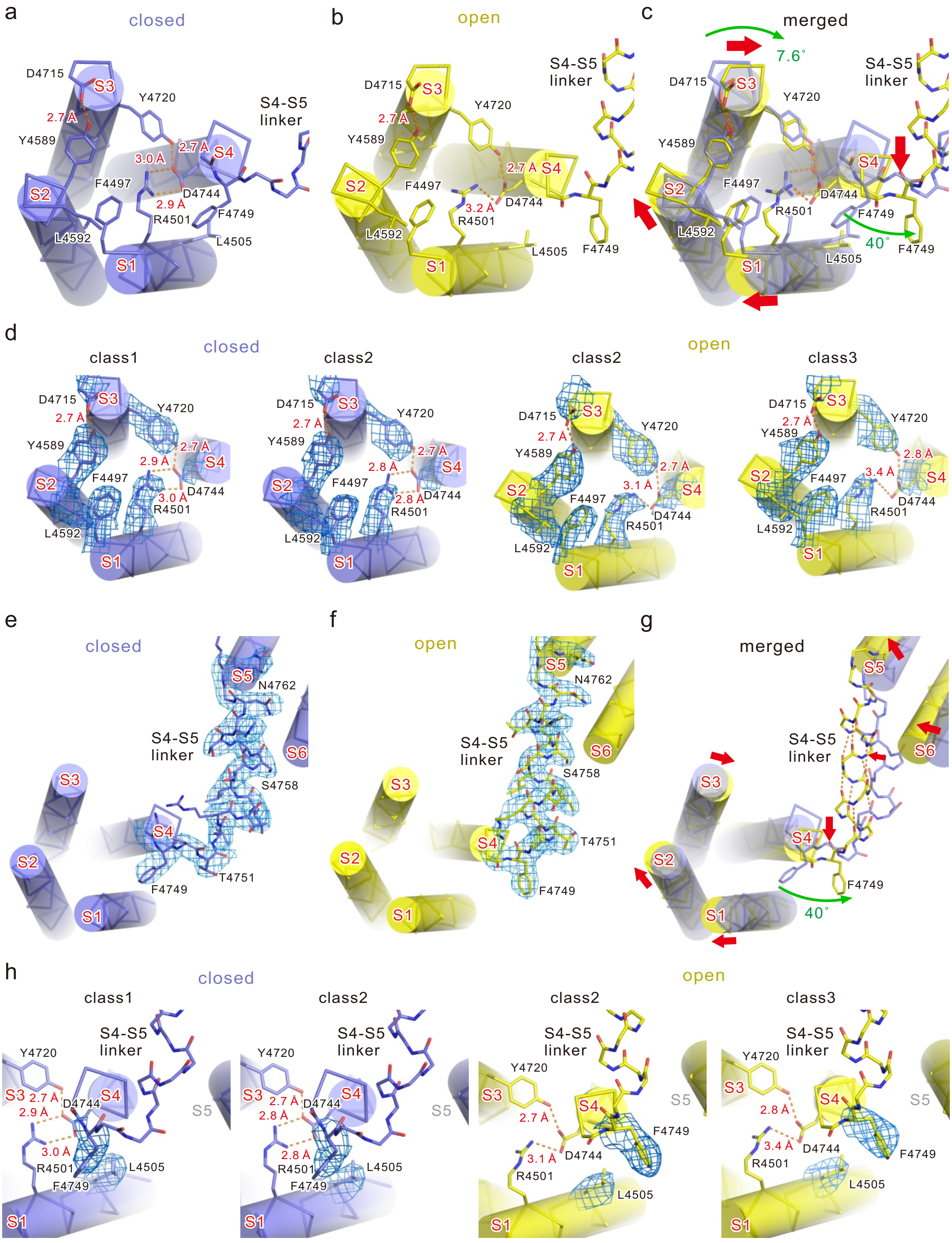

Supplementary Figure 4. Key interactions in the transmembrane region. a–c The S1-S4 bundle and part of S4-S5 linker. Closed state (**a**), open state (**b**), and overlay of the structures in both states (**c**) are shown as Cα models and overlaid with cylinder models. Viewed from the cytoplasm perpendicular to the membrane. Hydrogen bonds/salt bridges are shown as orange botted lines. **d** The S1-S4 bundle. Closed state and open state in the different classes in the same views shown in Fig. 3a and 3b. Density maps around side chains are superimposed and contoured at 0.03. **e-g** Drastic change in the S4-S5 linker upon binding of Ca^{2+}. The TM region in the closed (**e**) and open (**f**) states. Each density map around S4-S5 linker is superimposed and contoured at 0.03. (**g**) Overlay of the structures in both states (**g**), viewed from the cytoplasm perpendicular to the membrane, are shown as Cα models and overlaid with cylinder models. The S4-S5 linker did not form an α-helix in the closed state (**e**), but formed an α-helix in the open state (**f**). The color of carbon atoms is the same as that of Cα; oxygen, red; nitrogen, blue. Hydrogen bonds are shown as orange dotted lines. **h** TM region around S4. Closed state and open state in the different classes in the same view shown in Fig. 3h and 3i. Density maps around side chains are superimposed and contoured at 0.03.

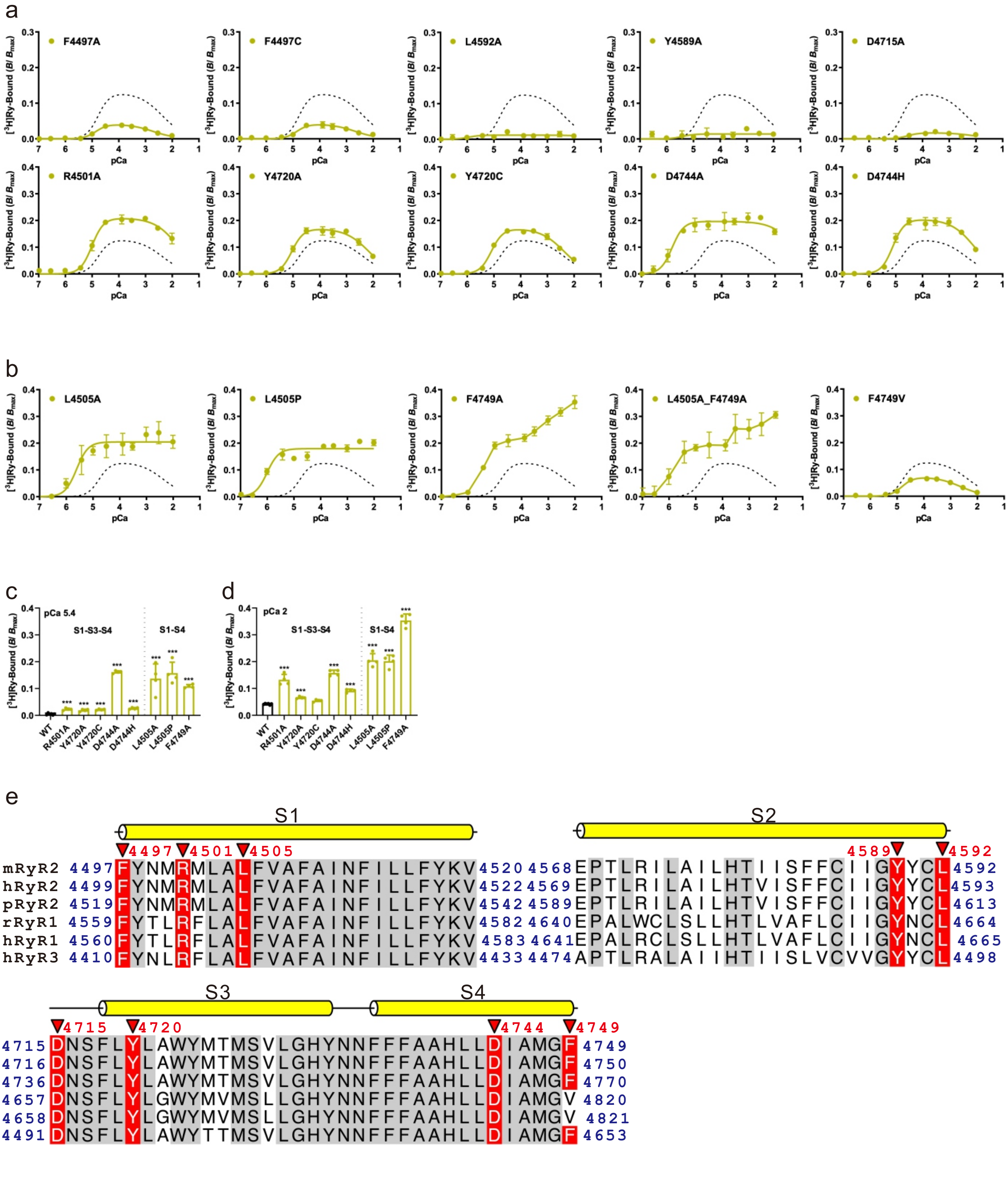

Supplementary Figure 5. Functional analysis of mutants involved in the movement of the TM region. a, **b** Ca^{2+}-dependent [^{3}H]ryanodine binding of WT (dotted line) and individual mutants involved in the S1/S2, S2/S3, S1/S3/S4 (a), or S1-S4 (b) interactions. Data are shown as means ± SD (n = 4). **c**, **d** [^{3}H]Ryanodine binding of WT and the mutants at pCa 5.4 (**c**) and pCa 2 (**d**). Data are shown as means ± SD (n = 4) and were analyzed by one-way ANOVA with Dunnett's test. ***p < 0.001 from WT. **e** Multiple sequence alignment of three RyR isoforms around S1, S2, and S3 to S4. The gray-shaded residues are the identical sequences among isoforms, and the residues for which the mutants were prepared and the functional assays were performed. Secondary structures are shown above the alignment. m, mouse; h, human; p, pig; r, rabbit. Source data are provided as a Source Dara file.

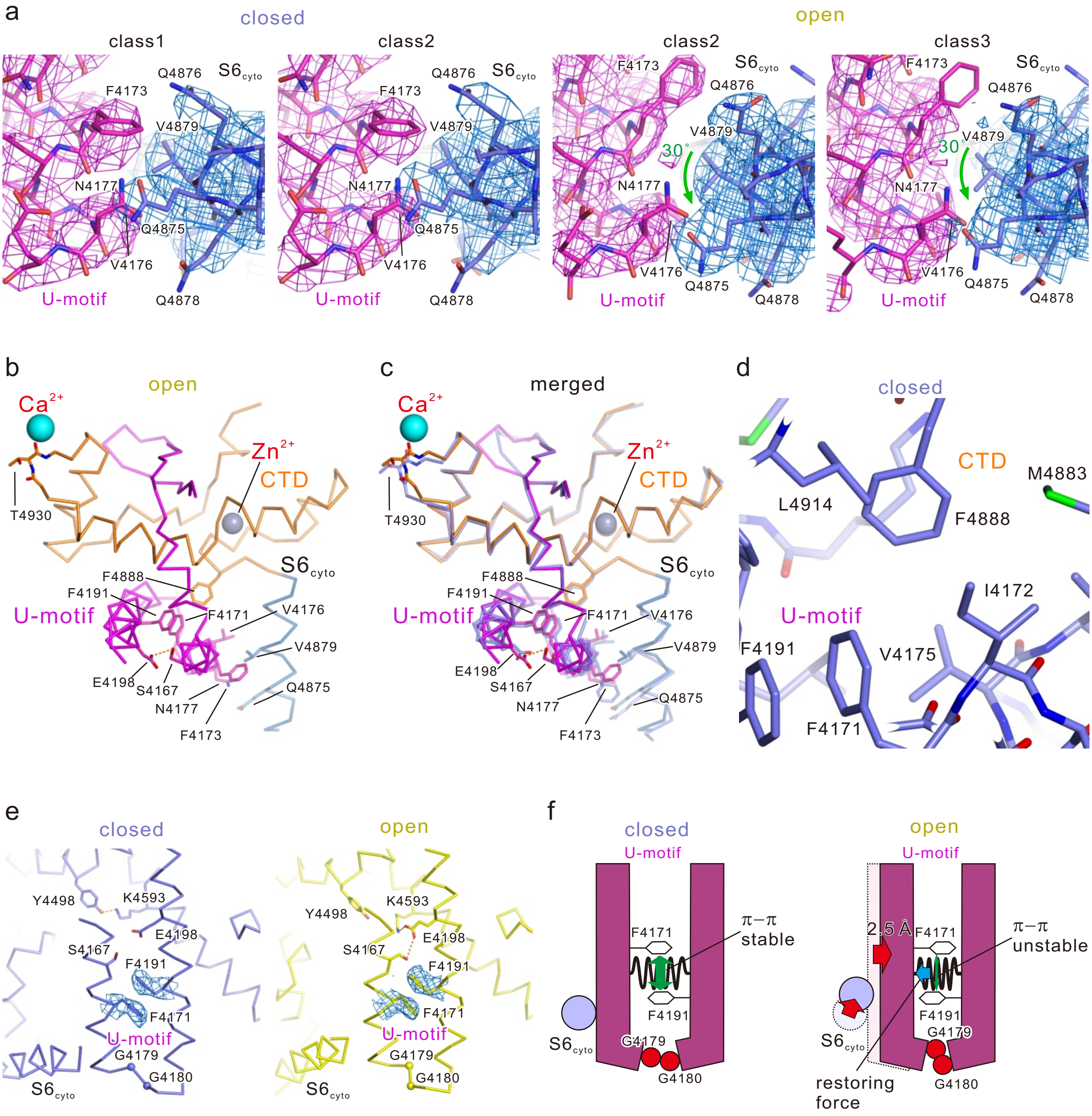

Supplementary Figure 6. Key interactions between the U-motif and S6$_{cyto}$/CTD. a Structures in the closed and open states in the different classes in the same views shown in in Fig. 4b and 4c. Density maps around the interaction are superimposed and contoured at 0.025. **b**, **c** Structure around the U-motif in the open state (**b**) and overlayed with the structure in the closed state (**c**) (U-motif, magenta; S6$_{cyto}$, blue; CTD, orange). Ca^{2+} and Zn^{2+} are shown as cyan and gray spheres, respectively. **d** Details of the U-motif/CTD interaction around F4888 in the closed state. **e** Density maps around the side chains of F4171 and F4191 in the closed state and open state contoured at 0.03. **f** Scheme of the compaction in U-motif and the critical role in the Interaction between F4171/F4191.

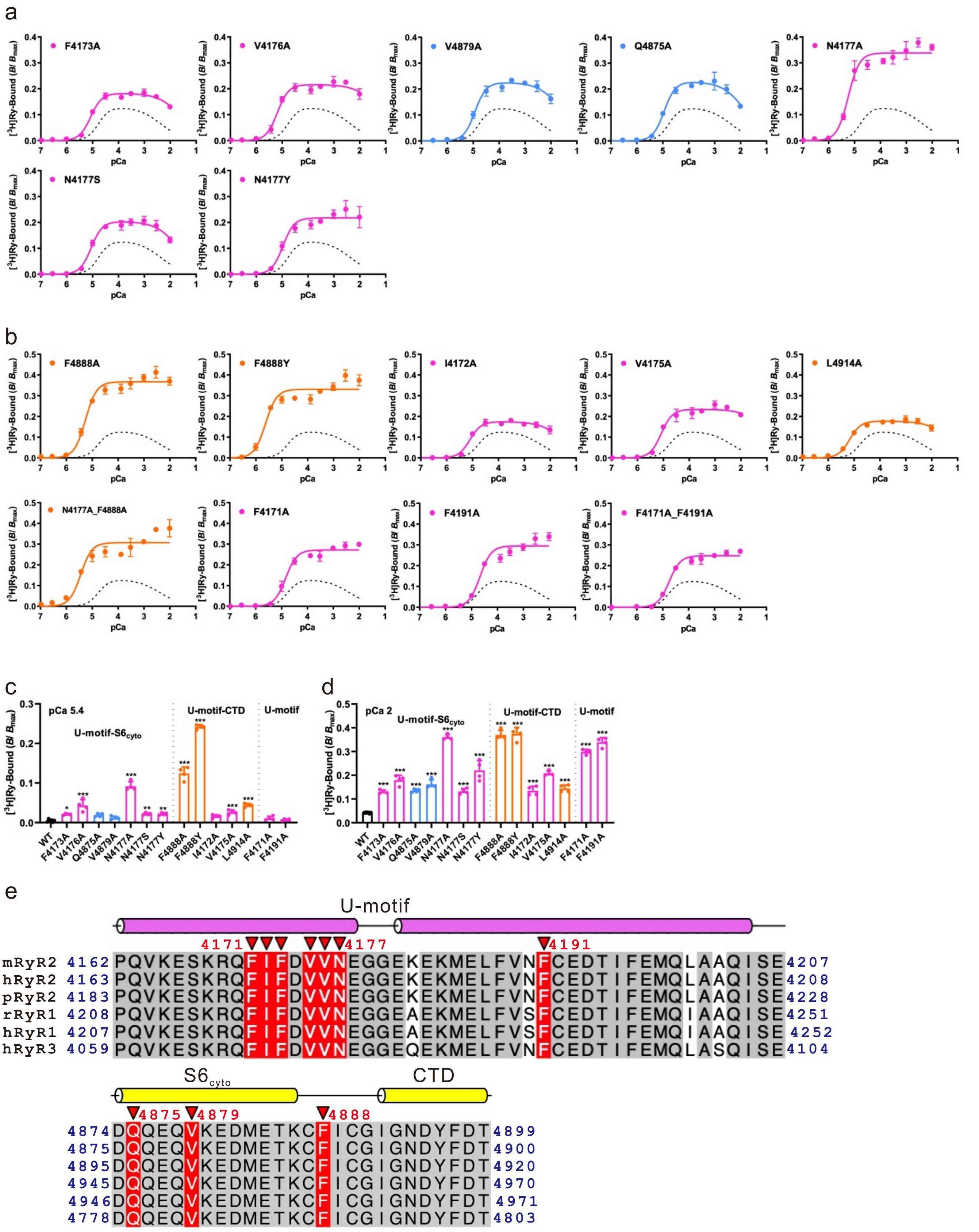

Supplementary Figure 7. Functional analysis of the mutants involved in the U-motif/ S6$_{cyto}$ and U-motif/CTD interactions. **a**, **b** Ca^{2+}-dependent [^{3}H]ryanodine binding of WT (dotted line) and individual mutants involved in the U-motif/S6$_{cyto}$ (**a**) and U-motif/CTD (**b**) interactions. Data are shown as means ± SD (n = 4). **c**, **d** [^{3}H]Ryanodine binding of WT and the mutants at pCa 5.4 (**c**) and pCa 2 (**d**). Data are shown as means ± SD (n = 4) and were analyzed by one-way ANOVA with Dunnett's test. *p < 0.05; **p < 0.01; ***p < 0.001. **e** Multiple sequence alignment of three RyR isoforms around the U-motif and S6$_{cyto}$ to the CTD. The gray-shaded residues are the identical sequences among isoforms, and the residues in which mutants were prepared and functional assayed are red-shaded. Secondary structures are shown above the alignment. m, mouse; h, human; p, pig; r, rabbit. Source data are provided as a Source Dara file.

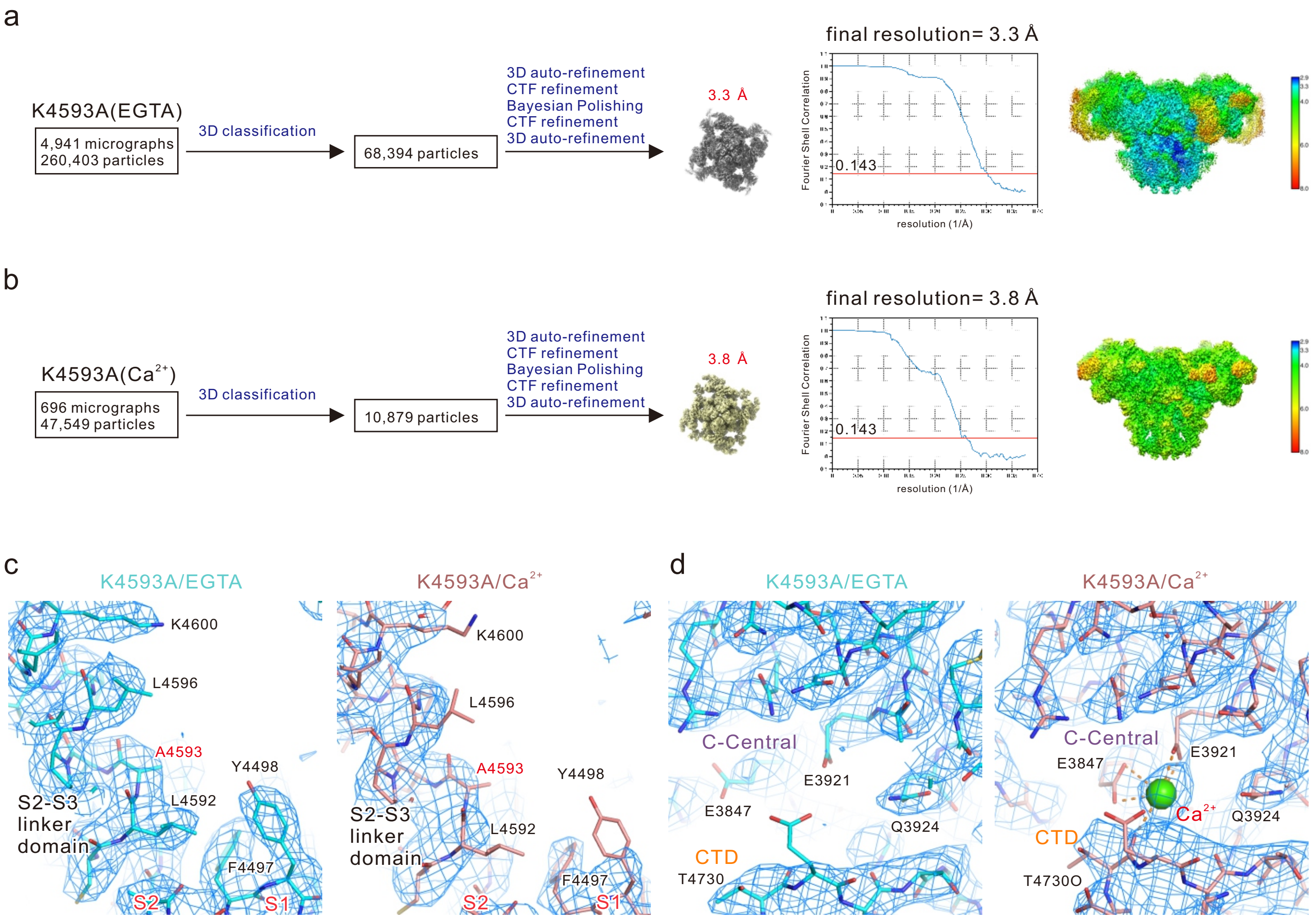

Supplementary Figure 8. Single particle analysis on the K4593A mutant. a, **b** Workflows for cryo-EM data processing and estimation of resolution and local resolution EM map of K4593A mutant in the presence of EGTA (K4593A(EGTA)) (**a**) and in the presence of Ca^{2+} (K4593A(Ca^{2+})) (**b**). **c**, **d** Density maps for the reconstructed structures of K4593A(EGTA) (left) and K4593A(Ca^{2+}) (right) around mutated residue (A4593) (**c**) and the Ca^{2+} binding site (**d**) with contour level at 0.035.

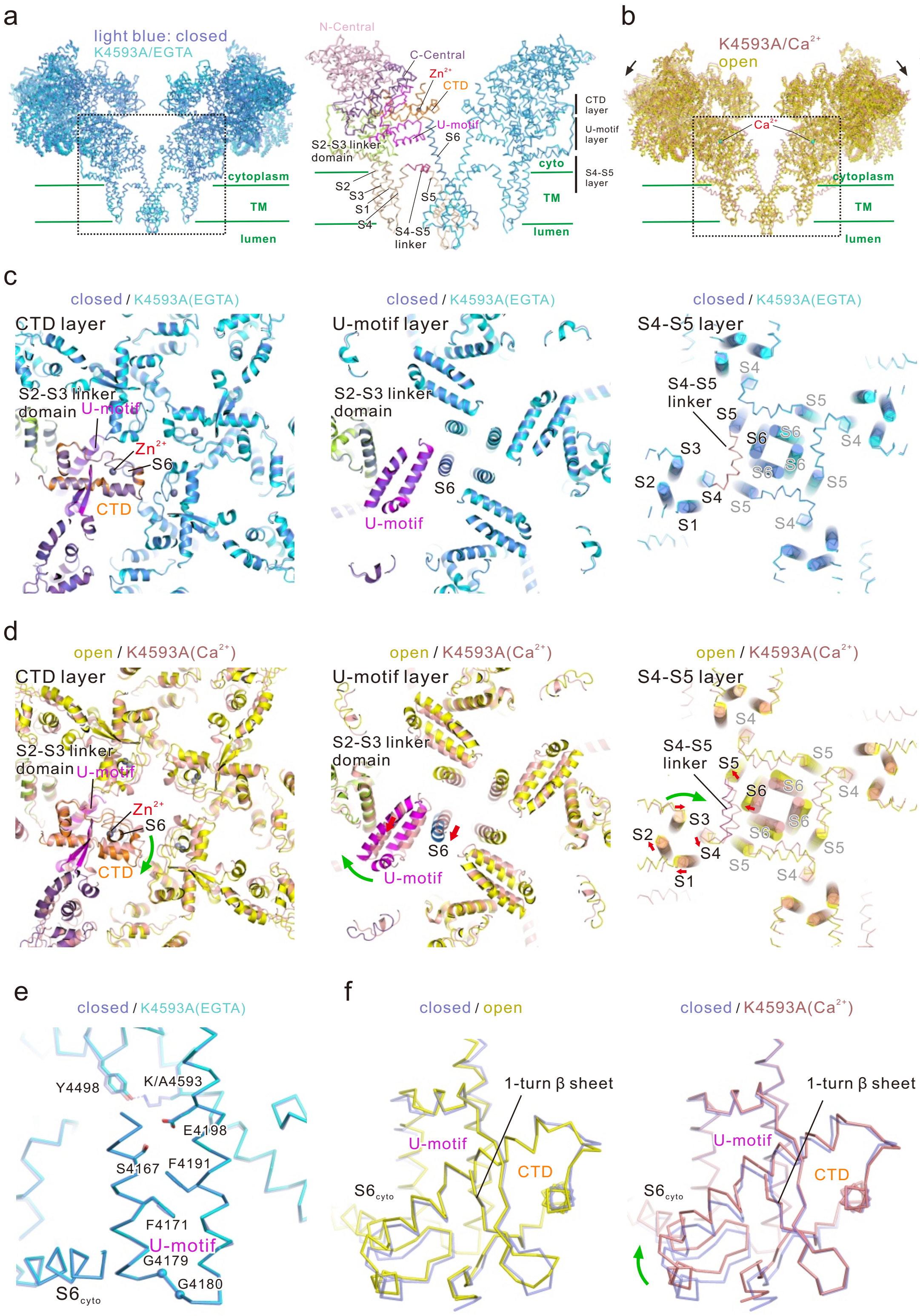

Supplementary Figure 9. Structures of the K4593A mutant. **a** Overlay of WT in the closed state shown in light blue and K4593A(EGTA) shown in cyan (left) and the magnified view of the dotted box in the left (right). **b** Overlay of K4593A(Ca^{2+}) shown in salmon and WT in the open state shown in yellow (left) and the magnified view of the dotted box in the left (right). **c**, **d** Cross-section views of CTD, U-motif, and S4-S5 layers. **c** Overlay of WT in the closed state (light blue) with K4593A(EGTA) (colored according to Fig. 1b or cyan). **d** Overlay of K4593A(Ca^{2+}) (salmon) with WT in the open state (colored according to Fig. 1b or yellow). **e** Analysis of the compaction in U-motif. Overlay of the structure in the closed state (light blue) and K4593A(EGTA) (cyan) are shown as a Cα model. The structures are fitted in the C-terminal side of U-motif (4183-4205). **f** Relationship between CTD, 1-turn beta sheet and U-motif. CTD is placed between 1-turn β sheet and U-motif. Left, overlay of the structure in the closed state (light blue) and in the open state (yellow) are shown as a Cα model. These regions in the both states matched well, suggesting that U-motif, CTD, and $S6_{cyto}$ rotated together upon Ca^{2+} binding. Right, overlay of the structure in the closed state (light blue) and K4593A(Ca^{2+}) (salmon) are shown as a Cα model. While U-motif and 1-turn β matched well, but CTD rotated clockwise independently (green arrow). The independent rotation of CTD in K4593A indicates that CTD placed between 1-turn β sheet and U-motif has some degree of freedom in the rotation in the K4593A mutant. These structures are fitted in the 1-turn β sheet and U-motif (4103-4203).

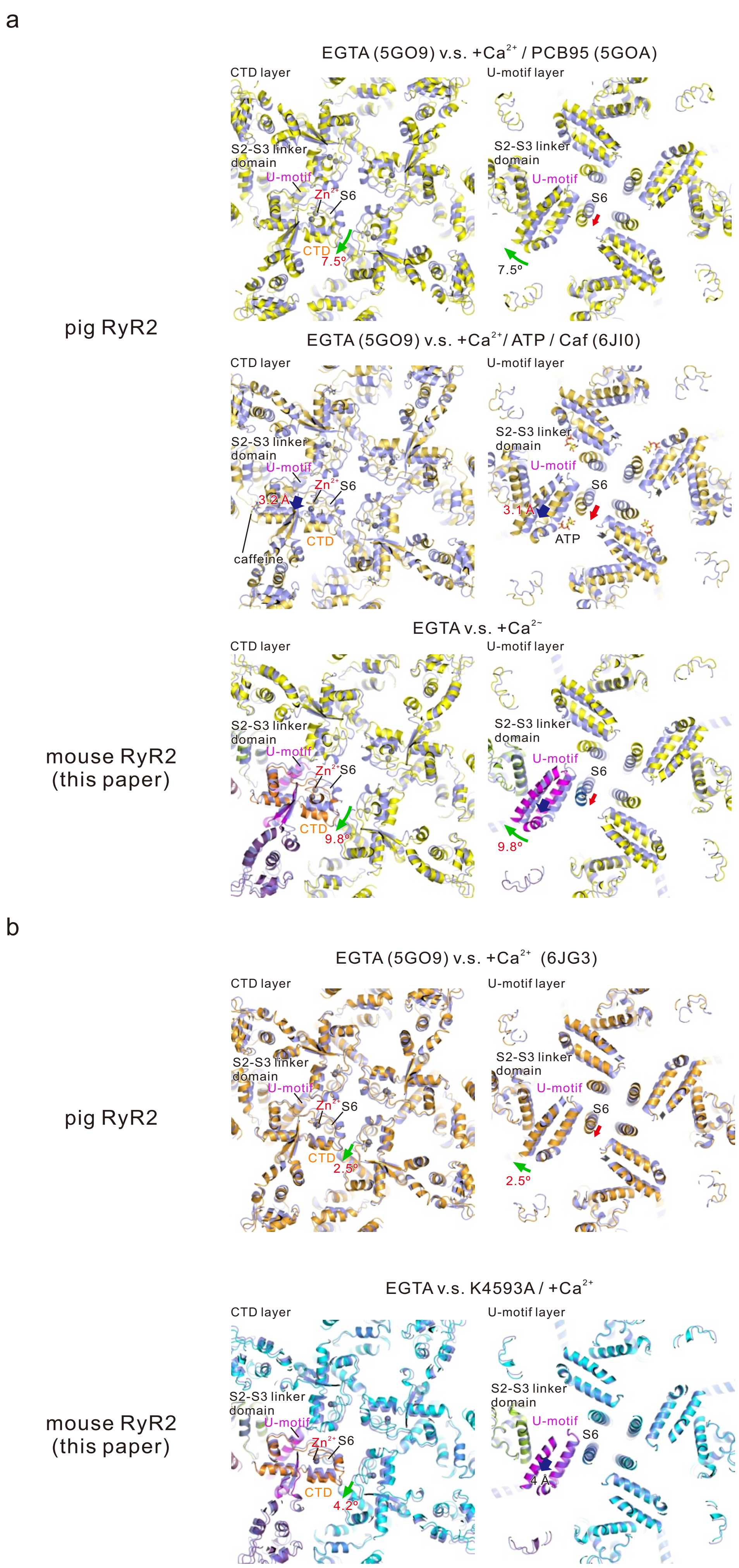
a
EGTA (5GO9) v.s. +Ca2+ / PCB95 (5GOA)
CTD layer
U-motif layer
S2-S3 linker domain
U-motif
Zn2+
S6
CTD
7.5º
pig RyR2
EGTA (5GO9) v.s. +Ca2+/ ATP / Caf (6JI0)
3.2 Å
3.1 Å
caffeine
ATP
EGTA v.s. +Ca2+
mouse RyR2 (this paper)
9.8º
b
EGTA (5GO9) v.s. +Ca2+ (6JG3)
2.5º
EGTA v.s. K4593A / +Ca2+
4.2º
4 Å

Supplementary Figure 10. Structural changes detected in previously reported structures. **a** Cross-section views of CTD and U-motif layers of pig RyR2 and mouse RyR2. Top and middle, the structure in the closed state (5GO9 [http://doi.org/10.2210/pdb5GO9/pdb], light blue) overlayed with structures in the open state with Ca^{2+}/PCB95 (5GOA [http://doi.org/10.2210/pdb5GOA/pdb], yellow) (top) and with Ca^{2+}/ATP/caffeine (6JI0 [http://doi.org/10.2210/pdb6JI0/pdb], yellow) (middel). Bottom, the structure of mouse RyR2 in the closed state (light blue) overlayed with the structure in the open state (colored according to Fig. 1b or yellow). **b** Cross-section views of CTD and U-motif layers of pig RyR2 and mouse RyR2. Top, the structure of pig RyR2 in the closed state (5GO9 [http://doi.org/10.2210/pdb5GO9/pdb], light blue) overlayed with the structure in the presence of Ca^{2+} (6JG3 [http://doi.org/10.2210/pdb6JG3/pdb], orange). Bottom, the structure of mouse K4593A(EGTA) (cyan) overlayed with the structure of mouse K4593A(Ca^{2+}) (colored according to Fig. 1b or salmon).

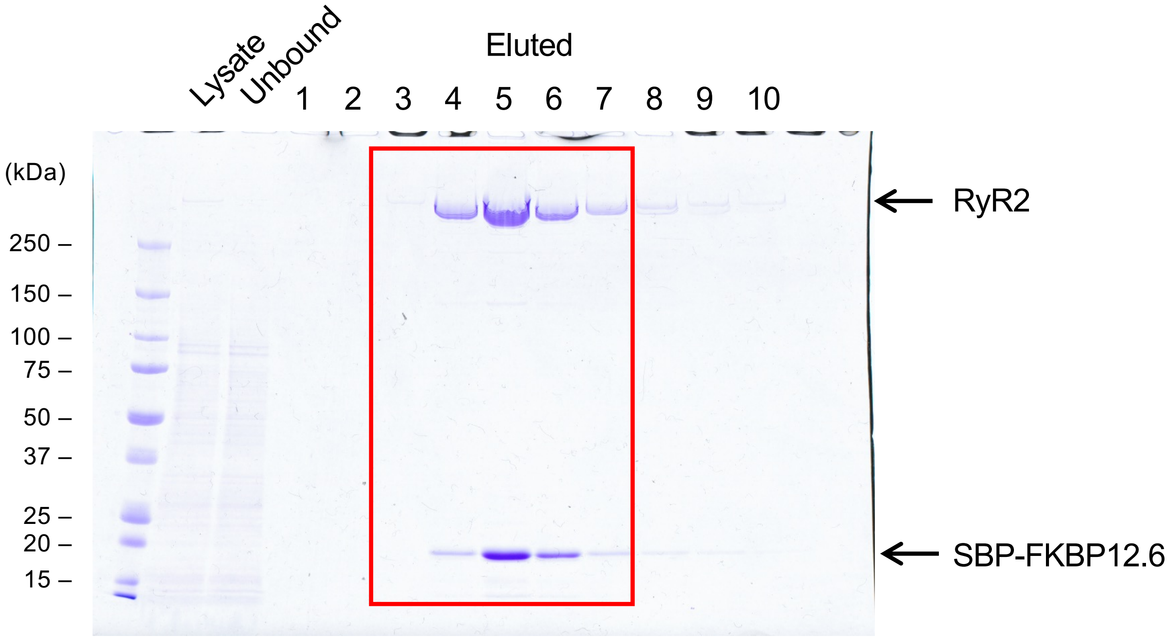

Supplementary Figure 11. Uncropped gel image for Supplementary Fig. 1a. Purification of SBP-FKBP12.6/RyR2 complex by StrepTrap column chromatography. Loaded lysate, unbound, and eluted fractions (1-10) were separated by SDS-PAGE. Area surrounded by red box was cut out and gray-scaled.

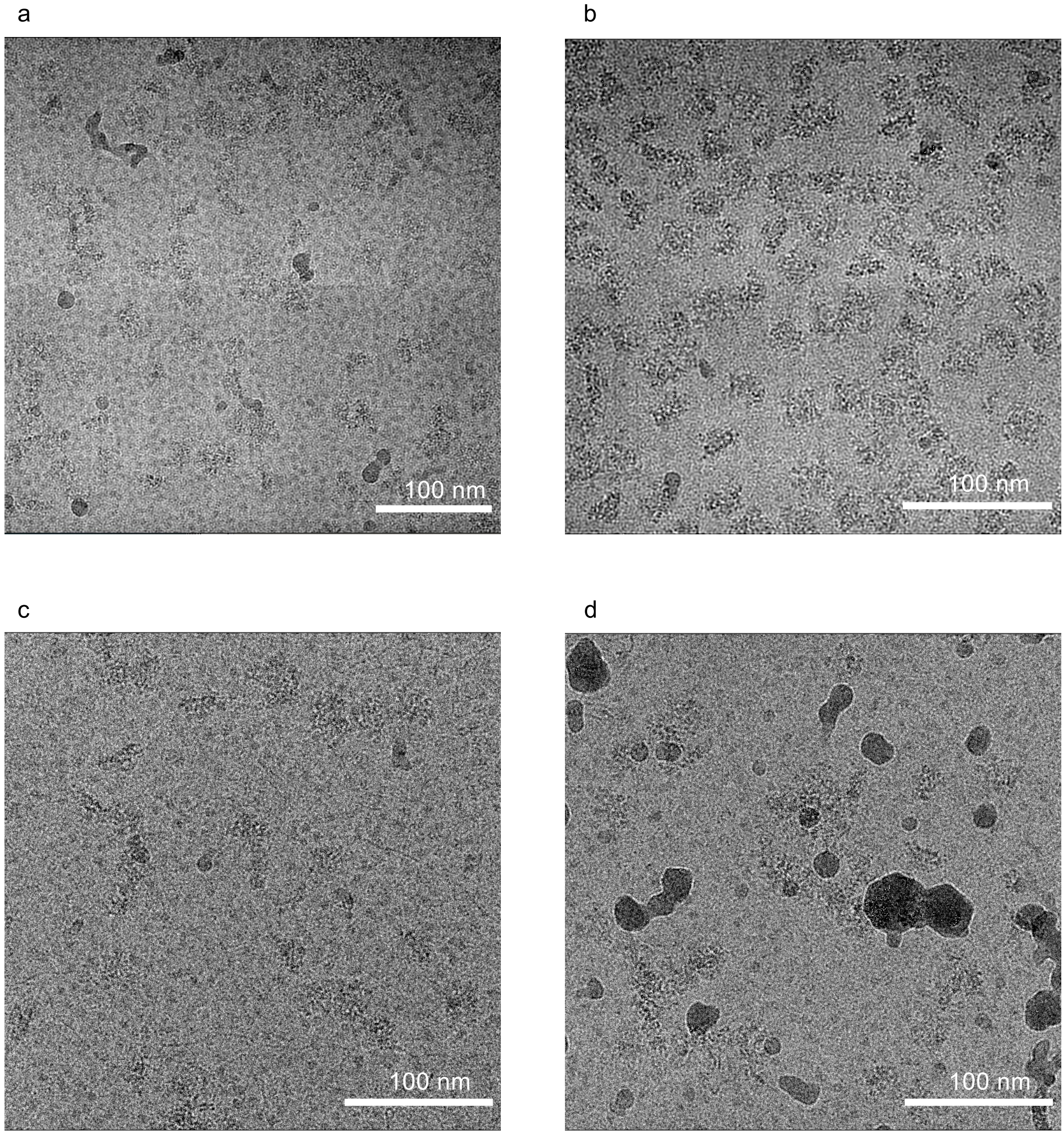

Supplementary Figure 12. Representative cryo-EM micrographs. a WT in the presence of EGTA, **b** WT in the presence of Ca^{2+}, **c** K4593A mutant in the presence of EGTA, **d** K4593A mutant in the presence of Ca^{2+}.

Supplementary Table 1. Data collection, rotamer outliers, processing, model refinement, and validation

Protein	WT						K4593A	
Condition	EGTA			100 µM Ca^{2+}			EGTA	100 µM Ca^{2+}
State	before classification	class1	class2	class1	class2	class3		
PBD ID	7VML	7VMM	7VMN	7VMO	7VMP	7VMQ	7VMR	7VMS
EMDB ID	EMD-30688	EMD-30689	EMD-30690	EMD-30691	EMD-30692	EMD-30693	EMD-32036	EMD-32037
**Data collection and processi**								
Microscope	Titan Krios G3i							
Detector	Gatan K3 BioQuantum							
Magnification	x81k			x105k			x105k	x105k
Voltage (kV)	300							
Electron exposure (e^{-}/Å^2)	50			60			50	50
Defocus range (µm)	0.5 - 2.0							
Pixcel size (Å)	1.07			0.83			0.83	
Symmetry imposed	*C* 4							
Initial particle images	578,868			388,457			260,403	47,549
Final particle images	109,425	45,120	41,197	45,432	42,375	40,665	68,394	10,879
Map resolution (Å)	3.3	3.49	3.52	3.45	3.51	3.72	3.3	3.8
FSC threshold	0.143	0.143	0.143	0.143	0.143	0.143	0.143	0.143
Map sharpening B-factor	-67	-22	-22	-49	-46	-65	-50	-84
**Model building and refineme**								
Model composition								
Protein atoms	123,564	123,564	123,564	122,032	122,032	122,032	123,548	123,548
Metals	4	4	4	8	8	8	4	8
R.M.S. deviations								
Bond length (Å)	0.002	0.002	0.003	0.002	0.003	0.003	0.003	0.003
Bond angles (°)	0.502	0.527	0.566	0.481	0.596	0.645	0.661	0.644
**Validation**								
MolProbity score	1.96	2.36	2.25	2.24	2.5	2.63	2.18	2.03
Clashscore								
whole domain	8.17	8.32	9.02	8.59	10.32	11.08	10.77	12.85
core domain	6.37	6.38	6.57	7.39	8.61	10.61	8.80	12.16
Rotamer outliers (%)	1.39	4.51	2.87	2.88	4.95	6.61	1.87	0.14
Ramachandran plot								
Favored (%)	93.85	93.8	93.71	93.37	93.18	93.14	93.59	93.8
Allowed (%)	6.15	6.2	6.29	6.63	6.82	6.86	6.41	6.2
Outlier (%)	0	0	0	0	0	0	0	0

Clashscores, rotamer outlers, and Ramachandran plots were calculated using PHENIX (Adams et al., 2010; Afonine et al., 2018).

Supplementary Table 2. RyR2 mutants used in this study.

Mutation	Domain	Disease	Ref	Ryanodine binding	ER Ca^{2+} level	Caffeine response	Functional change
S4167A	U-motif			↓	↑	±	LOF
S4167P	U-motif	CPVT, LQTS (RyR2)	1	↓	↑	±	LOF
F4171A	U-motif			↑	↓	+	GOF
I4172A	U-motif			↑	↓	+	GOF
F4173A	U-motif			↑	↓	+	GOF
V4175A	U-motif			↑	↓	+	GOF
V4176A	U-motif			↑	↓	+	GOF
N4177A	U-motif			↑	↓	+	GOF
N4177S	U-motif	CPVT (RyR2)	2	↑	↓	+	GOF
N4177Y	U-motif	CPVT (RyR2)	3	↑	↓	+	GOF
F4191A	U-motif			↑	↓	+	GOF
E4193A	U-motif			↓	↑	+	LOF
E4198A	U-motif			↓	↑	–	LOF
F4497A	S1			↓	↑	+	LOF
F4497C	S1	CPVT (RyR2)	4	↓	↑	+	LOF
Y4498A	S1			↓	↑	+	LOF
R4501A	S1			↑	↓	+	GOF
L4505A	S1			↑	↓	*	GOF
L4505P	S1	CCD (RyR1)	5	↑	↓	*	GOF
Y4589A	S2			↓	↑	+	LOF
L4592A	S2			↓	↑	+	LOF
K4593A	S2-S3			↓	↑	+	LOF
K4593Q	S2-S3	LQTS (RyR2)	6, 7	↓	↑	+	LOF
K4593R	S2-S3	IVF (RyR2)	8	↓	↑	+	LOF
R4607A	S2-S3			↓	↑	+	LOF
R4607Q	S2-S3	Sudden death (RyR2)	9	↓	↑	+	LOF
R4607W	S2-S3	Sudden death (RyR2)	10	↓	↑	+	LOF
D4715A	S3			↓	↑	+	LOF
Y4720A	S3			↑	↓	+	GOF
Y4720C	S3	CPVT (RyR2)	11	↑	↓	+	GOF
D4744A	S4			↑	↓	*	GOF
D4744H	S4	Myopathy (RyR1)	12	↑	↓	+	GOF
F4749A	S4			↑	↓	*	GOF
F4749V	S4			↓	↑	+	LOF
Q4875A	S6cyto			↑	↓	+	GOF
V4879A	S6cyto	CPVT (RyR2)	13	↑	↓	+	GOF
F4888A	CTD			↑	↓	+	GOF
F4888Y	CTD	MH/CCD (RyR1)	14	↑	↓	+	GOF
L4914A	CTD			↑	↓	+	GOF

CTD, C-terminal domain; CPVT, catecholaminergic polymorphic ventricular tachycardia; CCD, central core disease; LQTS, long QT syndrome; IVF, idiopathic ventricular fibrillation; MH, malignant hyperthermia; LOF, loss-of-function; GOF, gain-of-function. Caffeine response: +, >90% of cells were responded; ±, <50% of cells were responded; –, no cells were responded; *, no cells were responded due to ER Ca^{2+} depletion.

Supplementary References

1. Ozawa J, *et al.* Differential Diagnosis Between Catecholaminergic Polymorphic Ventricular Tachycardia and Long QT Syndrome Type 1- Modified Schwartz Score. *Circ J* **82**, 2269-2276 (2018).

2. Medeiros-Domingo A, *et al.* The RYR2-encoded ryanodine receptor/calcium release channel in patients diagnosed previously with either catecholaminergic polymorphic ventricular tachycardia or genotype negative, exercise-induced long QT syndrome: a comprehensive open reading frame mutational analysis. *J Am Coll Cardiol* **54**, 2065-2074 (2009).

3. Hayashi M, *et al.* Incidence and risk factors of arrhythmic events in catecholaminergic polymorphic ventricular tachycardia. *Circulation* **119**, 2426-2434 (2009).

4. Choi G, Kopplin LJ, Tester DJ, Will ML, Haglund CM, Ackerman MJ. Spectrum and frequency of cardiac channel defects in swimming-triggered arrhythmia syndromes. *Circulation* **110**, 2119-2124 (2004).

5. Wu S, *et al.* Central core disease is due to RYR1 mutations in more than 90% of patients. *Brain* **129**, 1470-1480 (2006).

6. Shigemizu D, *et al.* Exome analyses of long QT syndrome reveal candidate pathogenic mutations in calmodulin-interacting genes. *PLOS ONE* **10**, e0130329 (2015).

7. Hirose S, *et al.* Loss-of-Function Mutations in Cardiac Ryanodine Receptor Channel Cause Various Types of Arrhythmias Including Long QT Syndrome. *EP Europace* **in press**, (2021).

8. Paech C, Gebauer RA, Karstedt J, Marschall C, Bollmann A, Husser D. Ryanodine receptor mutations presenting as idiopathic ventricular fibrillation: a report on two novel familial compound mutations, c.6224T>C and c.13781A>G, with the clinical presentation of idiopathic ventricular fibrillation. *Pediatr Cardiol* **35**, 1437-1441 (2014).

9. Wong LC, Roses-Noguer F, Till JA, Behr ER. Cardiac evaluation of pediatric relatives in sudden arrhythmic death syndrome: a 2-center experience. *Circ Arrhythm Electrophysiol* **7**, 800-806 (2014).

10. Wang D, *et al.* Cardiac channelopathy testing in 274 ethnically diverse sudden unexplained deaths. *Forensic Sci Int* **237**, 90-99 (2014).

11. Roston TM, *et al.* Catecholaminergic polymorphic ventricular tachycardia patients with multiple genetic variants in the PACES CPVT Registry. *PLOS ONE* **13**, e0205925 (2018).

12. Bharucha-Goebel DX, *et al.* Severe congenital RYR1-associated myopathy: the expanding clinicopathologic and genetic spectrum. *Neurology* **80**, 1584-1589 (2013).

13. Bagattin A, *et al.* Denaturing HPLC-based approach for detecting RYR2 mutations involved in malignant arrhythmias. *Clin Chem* **50**, 1148-1155 (2004).

14. Ibarra M CA, *et al.* Malignant hyperthermia in Japan: mutation screening of the entire ryanodine receptor type 1 gene coding region by direct sequencing. *Anesthesiology* **104**, 1146-1154 (2006).
