# Supplementary material for: Molecular basis for gating of cardiac ryanodine receptor explains the mechanisms for gain- and loss-of function mutations
Source: Nat Commun. 2022 May 20;13:2821. doi: 10.1038/s41467-022-30429-x (PMC9123176; doi:10.1038/s41467-022-30429-x)
Supplement: Supplementary file 3 — Description of Additional Supplementary Files [file 41467_2022_30429_MOESM3_ESM.pdf]

File name: Supplementary Movie 1

Description: The architecture of RyR2 and details of its opening.

File name: Supplementary Movie 2

Description: Rotation of C-Central/U-motif/U-motif/$S6_{cyto}$/CTD complex upon Ca^{2+} binding.

File name: Supplementary Movie 3

Description: Details of signal transmission from the cytoplasm into the TM region via the U-motif/S2-S3 linker domain interaction.

File name: Supplementary Movie 4

Description: Conformational changes in the TM region upon Ca^{2+} binding.

File name: Supplementary Movie 5

Description: Details of the U-motif/$S6_{cyto}$/CTD interaction.

File name: Supplementary Movie 6

Description: Structure comparison of the K4593A mutant with the WT in the closed state and open state.
