# Supplementary material for: Molecular basis for gating of cardiac ryanodine receptor explains the mechanisms for gain- and loss-of function mutations
Source: Nat Commun. 2022 May 20;13:2821. doi: 10.1038/s41467-022-30429-x (PMC9123176; doi:10.1038/s41467-022-30429-x)
Supplement: Supplementary file 10 — Reporting Summary [file 41467_2022_30429_MOESM10_ESM.pdf]

nature portfolio

Corresponding author(s): Haruo Ogawa
Last updated by author(s): Apr 4, 2022

Reporting Summary

Nature Portfolio wishes to improve the reproducibility of the work that we publish. This form provides structure for consistency and transparency in reporting. For further information on Nature Portfolio policies, see our Editorial Policies and the Editorial Policy Checklist.

Statistics

For all statistical analyses, confirm that the following items are present in the figure legend, table legend, main text, or Methods section.

n/a	Confirmed	
☐	☒	The exact sample size (*n*) for each experimental group/condition, given as a discrete number and unit of measurement
☐	☒	A statement on whether measurements were taken from distinct samples or whether the same sample was measured repeatedly
☐	☒	The statistical test(s) used AND whether they are one- or two-sided<br>*Only common tests should be described solely by name; describe more complex techniques in the Methods section.*
☒	☐	A description of all covariates tested
☐	☒	A description of any assumptions or corrections, such as tests of normality and adjustment for multiple comparisons
☐	☒	A full description of the statistical parameters including central tendency (e.g. means) or other basic estimates (e.g. regression coefficient) AND variation (e.g. standard deviation) or associated estimates of uncertainty (e.g. confidence intervals)
☐	☒	For null hypothesis testing, the test statistic (e.g. *F*, *t*, *r*) with confidence intervals, effect sizes, degrees of freedom and *P* value noted<br>*Give P values as exact values whenever suitable.*
☐	☒	For Bayesian analysis, information on the choice of priors and Markov chain Monte Carlo settings
☒	☐	For hierarchical and complex designs, identification of the appropriate level for tests and full reporting of outcomes
☒	☐	Estimates of effect sizes (e.g. Cohen's *d*, Pearson's *r*), indicating how they were calculated

Our web collection on statistics for biologists contains articles on many of the points above.

Software and code

Policy information about availability of computer code

Data collection	SerialEM 3.7.10 was used to collect movie stacks for cryo-EM.
Data analysis	CTFFIND4, RELION 3.0, RELION 3.1, CNS 1.3, PHENIX 1.19, COOT 0.8.9.2, PyMOL v2.4.0, UCSF Chimera 1.14, HOLE 2, GraphPad Prism 9, WEBMAXC STANDARD (a program on the web with no version information) were used to analyze the data in this study.

For manuscripts utilizing custom algorithms or software that are central to the research but not yet described in published literature, software must be made available to editors and reviewers. We strongly encourage code deposition in a community repository (e.g. GitHub). See the Nature Portfolio guidelines for submitting code & software for further information.

Data

Policy information about availability of data

All manuscripts must include a data availability statement. This statement should provide the following information, where applicable:

- Accession codes, unique identifiers, or web links for publicly available datasets
- A description of any restrictions on data availability
- For clinical datasets or third party data, please ensure that the statement adheres to our policy

Atomic coordinates and cryo-EM density maps have been deposited in the Protein Data Bank (PDB) and the Electron Microscopy Data Bank (EMDB) under the accession codes 7VML [http://doi.org/10.2210/pdb7VML/pdb] and EMD-30688 [https://www.ebi.ac.uk/pdbe/entry/emdb/EMD-30688] (closed state before classification), 7VMM [http://doi.org/10.2210/pdb7VMM/pdb] and EMD-30689 [https://www.ebi.ac.uk/pdbe/entry/emdb/EMD-30689] (closed state class1), 7VMN [http://doi.org/10.2210/pdb7VMN/pdb] and EMD-30690 [https://www.ebi.ac.uk/pdbe/entry/emdb/EMD-30690] (closed state class2), 7VMO [http://doi.org/10.2210/pdb7VMO/pdb] and EMD-30691 [https://www.ebi.ac.uk/pdbe/entry/emdb/EMD-30691] (open state class1), 7VMP [http://doi.org/10.2210/pdb7VMP/pdb] and EMD-30692 [https://www.ebi.ac.uk/pdbe/entry/emdb/EMD-30692] (open state class2), 7VMQ [http://doi.org/10.2210/pdb7VMQ/pdb] and

EMD-30693 [https://www.ebi.ac.uk/pdbe/entry/emdb/EMD-30693] (open state class3), 7VMR [http://doi.org/10.2210/pdb7VMR/pdb] and EMD-32036 [https://www.ebi.ac.uk/pdbe/entry/emdb/EMD-32036] (K4593A mutant in the presence of 1 mM EGTA), 7VMS [http://doi.org/10.2210/pdb7VMS/pdb] and EMD-32037 [https://www.ebi.ac.uk/pdbe/entry/emdb/EMD-32037] (K4593A mutant in the presence of 100 µM of Ca2+).

Field-specific reporting

Please select the one below that is the best fit for your research. If you are not sure, read the appropriate sections before making your selection.

☒ Life sciences ☐ Behavioural & social sciences ☐ Ecological, evolutionary & environmental sciences

For a reference copy of the document with all sections, see nature.com/documents/nr-reporting-summary-flat.pdf

Life sciences study design

All studies must disclose on these points even when the disclosure is negative.

Sample size	The sample size for each experiment was determined based on pilot experiments, which provided an estimate of the effect size and of data variability.
Data exclusions	No data were excluded in this study.
Replication	All functional measurements were repeated at least three times independently over the past three years. All attempts at replication were successful and produced similar results.
Randomization	In this study, randomization was not performed, because allocation was not performed.
Blinding	In this study, blinding was not performed, because allocation was not performed.

Reporting for specific materials, systems and methods

We require information from authors about some types of materials, experimental systems and methods used in many studies. Here, indicate whether each material, system or method listed is relevant to your study. If you are not sure if a list item applies to your research, read the appropriate section before selecting a response.

Materials & experimental systems

n/a	Involved in the study	
☒	☐	Antibodies
☐	☒	Eukaryotic cell lines
☒	☐	Palaeontology and archaeology
☒	☐	Animals and other organisms
☒	☐	Human research participants
☒	☐	Clinical data
☒	☐	Dual use research of concern

Methods

n/a	Involved in the study	
☒	☐	ChIP-seq
☒	☐	Flow cytometry
☒	☐	MRI-based neuroimaging

Eukaryotic cell lines

Policy information about cell lines

Cell line source(s)	We used Flp-In T-REX293 cell lines from LifeTechnologies.
Authentication	None of cell lines used have been authenticated.
Mycoplasma contamination	We did not test for mycoplasma contamination.
Commonly misidentified lines (See ICLAC register)	No commonly misidentified lines were used in this study.
